# Rheological and Antimicrobial Properties of Chitosan and Quinoa Protein Filmogenic Suspensions with Thyme and Rosemary Essential Oils

**DOI:** 10.3390/foods9111616

**Published:** 2020-11-06

**Authors:** Monserrat Escamilla-García, Raquel A. Ríos-Romo, Armando Melgarejo-Mancilla, Mayra Díaz-Ramírez, Hilda M. Hernández-Hernández, Aldo Amaro-Reyes, Prospero Di Pierro, Carlos Regalado-González

**Affiliations:** 1 Department of Food Research and Postgraduate Studies, Faculty of Chemistry, Autonomous University of Querétaro, C.U., Cerro de las Campanas S/N, Col. Las Campanas, Querétaro 76010, Mexico; moneg14@hotmail.com (M.E.-G.); riosromo1@gmail.com (R.A.R.-R.); armema2410@gmail.com (A.M.-M.); aldoamaro@gmail.com (A.A.-R.); 2 Department of Food Science, Division of Biological Sciences and Health, Autonomous Metropolitan University, Lerma Unit, Avenida de las Garzas N°. 10, El Panteón, Lerma de Villada 52005, Mexico; marea131079@gmail.com; 3 CONACyT-Center for Research Technological Assistance and Design of the State of Jalisco, A.C. (CIATEJ), Av. Normalistas 800, Volinas de la Normal, Guadalajara 44270, Jalisco, Mexico; hhernandez@ciatej.mx; 4 Department of Chemical Sciences, University of Naples “Federico II”, 80126 Naples, Italy; prospero.dipierro@unina.it

**Keywords:** filmogenic suspension, *Salmonella*, thyme, rosemary

## Abstract

Food packaging faces the negative impact of synthetic materials on the environment, and edible coatings offer one alternative from filmogenic suspensions (FS). In this work, an active edible FS based on chitosan (C) and quinoa protein (QP) cross-linked with transglutaminase was produced. Thyme (T) and rosemary (R) essential oils (EOs) were incorporated as antimicrobial agents. Particle size, Z potential, and rheological parameters were evaluated. The antimicrobial activity against *Micrococcus luteus* (NCIB 8166) and *Salmonella* sp. (Lignieres 1900) was monitored using atomic force microscopy and image analysis. Results indicate that EOs incorporation into C:QP suspensions did not affect the Z potential, ranging from −46.69 ± 3.19 mV to −46.21 ± 3.83 mV. However, the polydispersity index increased from 0.51 ± 0.07 to 0.80 ± 0.04 in suspensions with EO. The minimum inhibitory concentration of active suspensions against *Salmonella* sp. was 0.5% (*v*/*v*) for thyme and 1% (*v*/*v*) for rosemary. Entropy and fractal dimension of the images were used to confirm the antimicrobial effect of EOs, which modified the surface roughness.

## 1. Introduction

Numerous factors affect the original quality of food products [1], and up to date, many synthetic food packaging materials are used due to their good mechanical and barrier properties, but they show long biodegradation processes [2]. Besides providing food protection from the environment, active packaging may also protect from foodborne illness outbreaks [3,4].

Biopolymers are commonly used to produce coatings, such as polysaccharides (starch, chitosan, cellulose), proteins (animal or vegetable), and also lipids (waxes, fatty acids), or a mixture of them [4]. These materials act as barriers against the transport of gases and water vapor, leading to longer shelf life, keeping the organoleptic properties of foods. Protection from spoilage and pathogenic microorganisms may be achieved by incorporating antimicrobial compounds [5].

Coatings made from filmogenic suspensions (FS) of polysaccharides are primarily designed to be an efficient oxygen barrier due to their well-ordered hydrogen-bonded network. However, they provide a deficient moisture barrier due to their hydrophilic nature. Polysaccharide coatings are colorless, show good appearance, and alone or in combination with other biopolymers may be used to extend the shelf life of fruits, vegetables, seafood, or meat products by significantly reducing dehydration, surface darkening, and rancidity [6]. Chitosan (C) is the second most abundant polysaccharide in nature, comprising two units β-(1-4)-2-acetamido-d-glucose, and β-(1-4)-2-amino-d-glucose [7]. Chitosan is described in terms of deacetylation extent and average molecular weight. This compound’s importance relies on antimicrobial properties, together with its cationic nature and film-forming properties [8]. Chitosan-based coatings show extremely low oxygen permeability, low relative humidity, and high water vapor permeability [9]. Chitosan exhibits bacteriostatic and fungistatic properties, and thus, can be used for active packaging, producing films of good mechanical properties, high permeability to CO_2_, and low to O_2_ [10].

FS of proteins may produce coatings by their denaturation using heat, solvents, or pH changes, followed by association of peptide chains through new intermolecular interactions [7]. The polymeric interactions produce coatings with a rigid protein network, less flexible, and less permeable to gases and vapors. Therefore, protein-based films or coatings are considered highly effective oxygen barriers, even at high relative humidity [11]. There are limited reports on FS of quinoa protein (*Chenopodium quinoa* Willd; QP), despite being able to produce edible coatings that combined with chitosan showed enhanced mechanical properties [12]; while the addition of small amounts of plasticizers to this mixture results in improved water vapor permeability [13].

Different antimicrobial agents have been added to edible coatings to avoid microbial contamination in food [14]. Essential oils are aromatic compounds of natural origin, with a broad spectrum of biological activities [15], and many exert strong antibacterial, antiviral and antifungal activities, leading to wide applications in food and beverage products [16].

Thyme (*Thymus vulgaris*) (T) essential oil (EO) has been used as a flavor ingredient in a wide variety of foods, beverages, and confectionery. It has been labeled as Generally Recognized as Safe (GRAS) food additive by the Food and Drug Administration (FDA) of the USA [17]. The antimicrobial activity is mainly due to thymol and carvacrol, which are also found in other EOs. Its antioxidant properties have been used to combat reactive oxygen species and prevent oxidation of food [18]. This oil has demonstrated antifungal activity against *Aspergillus*, *Penicillium*, *Ulocladium*, *Cladosporium*, *Trichoderma*, *Rhizopus*, and *Chaetomium* [19]. Thyme spectrum against pathogenic bacteria includes *Listeria monocytogenes*, *Pseudomonas aeruginosa*, *Staphylococcus aureus*, and *Salmonella* sp. [20,21].

Rosemary (*Rosmarinus officinalis* L. Labiadas) is a long-lasting aromatic perennial herb, and its oil has been used in food preservation [22]. Rosemary EO contains monoterpenes such as 1,8-cineole, α-pinene, camphor, and camphene, which give antioxidant, antimicrobial, and anticancer effects [23]. Its effect has been analyzed against Gram-positive (*S. aureus* and *Bacillus subtilis*) and Gram-negative (*Escherichia coli*, *Salmonella enteritidis*, and *Klebsiella pneumoniae*) strains. However, it has shown greater activity against Gram-positive bacteria [24]. This EO has been studied as an active agent in cellulose acetate films or chitosan coatings for food preservation [25]. In this work, we demonstrate the antimicrobial effect of rosemary and thyme EOs incorporated into QP:C filmogenic suspensions on Salmonella sp.’s surface microstructure, which has been reported as the second largest responsible for food outbreaks in the USA [26]. Few reports perform texture image analysis from atomic force micrographs to determine image texture, fractal dimension, and roughness, to visualize the antimicrobial effect of the active FS against this pathogen. This has not been previously reported for these active filmogenic suspensions. Here we show the effect of the FS containing R and T EOs on microbial population reduction using conventional methodology, which is compared with high resolution visualization of their mechanisms of action at cell surface level by atomic force microscopy, which to our knowledge has not been previously reported.

The present work’s objective was to develop and characterize a filmogenic suspension based on chitosan and quinoa protein cross-linked with transglutaminase, with antimicrobial effect by incorporation of rosemary and thyme essential oils.

## 2. Materials and Methods

### 2.1. Supplies

Chitosan (Cat. No. 417963, Sigma-Aldrich, St. Louis, MO, USA), Peruvian commercial quinoa (Hanseatik), rosemary (R) EO (Drogueria Cosmopolita, Ciudad de México, Mexico), thyme (T) EO (Drogueria Cosmopolita, CDMX, Mexico), sorbitol (Cat. No. W302902, Sigma Aldrich), microbial transglutaminase (TG) derived from *Streptoverticillium* sp., with 92 IU/g (Activa WM, Ajinomoto, France). *Salmonella* sp. and *Micrococcus luteus* NCIB 8166 were obtained from the Department of Food Research and Postgraduate Studies of the Autonomous University of Querétaro, Querétaro, México. Tryptic soy broth (TSB) was purchased from BD Difco (Franklin Lakes, NJ, USA).

### 2.2. Methods

#### 2.2.1. Quinoa Protein Extraction

The quinoa seeds were ground to about 200 µm using a coffee grinder (Krups Model GX410011, Solingen, Germany). The flour was defatted by three extractions with ethanol (70% *v*/*v*) in the ratio 1:10 *w*/*v* (flour:solvent) under constant stirring for 2 h and 25 °C [27,28]. The defatted flour was suspended in distilled water (10%, *w*/*v*) adjusting to pH 11 with 1 N NaOH, and stirred for 1 h at room temperature (25 °C). Then, the samples were centrifuged at 3200× *g*, at 10 °C for 30 min. The supernatant was adjusted to pH 4.5 and stirred for 30 min, followed by centrifugation as before. The precipitate was re-suspended in distilled water at a 5:95 ratio (precipitate: water, *w*/*v*) and neutralized with 2 N NaOH, followed by oven drying at 50 °C (ED, Binder, Tuttlingen, Germany). The protein isolates were ground for 2 min using the coffee grinder and passed through a No. 9 mesh (Tyler standard) of 200 μm pore opening [12].

#### 2.2.2. Quinoa Protein-Chitosan Filmogenic Suspension

The quinoa protein (QP) FS (2% *w*/*v*) was adjusted to pH 11, with constant stirring for 1 h. On the other hand, a 2% (*w*/*v*) chitosan solution was prepared in 0.5 M HCl, according to Escamilla [12]. The solutions were then mixed in 1:10 ratio (C:QP), sorbitol was added in a 1:1 weight ratio (C:sorbitol), and adjusted to pH 11. Then, the mixture was homogenized by a high speed mixer (IKA T25-Ultra-Turrax, Wilmington, USA) at 21,500 rpm for 3 min, followed by sonication for 10 min at 150 W, and 20 kHz (SONOPULS, HD3200, Bandelin GmbH, Berlin, Germany) [29].

#### 2.2.3. Crosslinking with Transglutaminase

The C:QP FS (Section 2.2.2) before homogenization was adjusted to pH 9 and added with 1.4% (*v*/*v*) of TG solution (10% *w*/*v*), stirred for 1 h, followed by pH adjustment to 11. After cross-linking, the FS was homogenized by a high-speed mixer (IKA T25-Ultra-Turrax) at 21,500 rpm for 3 min, followed by sonication for 10 min at 150 W and 20 kHz (SONOPULS, HD3200).

#### 2.2.4. Minimum Inhibitory Concentration

The minimum inhibitory concentration (MIC) of the EOs was evaluated against *Salmonella* sp., and *Micrococcus luteus*, adjusted to an optical density of 0.08 (10^6^ CFU/mL) at 600 nm. *Salmonella* sp. was chosen due to its highly frequent presence in foodborne outbreaks, being lethal in many cases. *Micrococcus luteus* is not a severe pathogenic bacterium, but for many years it has been used as a model system for bacterial cell wall study due to low peptidoglycan cross-linking (about 25%) [30,31]. *M. luteus* sensitivity to cell wall disruption was the reason to choose this microorganism as Gram-positive model. EOs at six different concentrations (0%, 0.5%, 0.7%, 1.0%, 1.5%, and 2.0%, *v*/*v*), and Tween 80 at constant concentration of 0.5% (*v*/*v*), were added to the TSB culture media. Absorbance readings were recorded every h, and the MIC was considered as the lowest concentration tested that inhibited microbial growth.

#### 2.2.5. Active Filmogenic Suspension

The 1:10 C:QP FS (Section 2.2.2) was cross-linked with TG (Section 2.2.3), and then the active FS was produced by adding the EOs at the previously found MIC and Tween 80 at 0.5% (*v*/*v*). The active FS was homogenized by a high-speed mixer (IKA T25-Ultra-Turrax) at 21,500 rpm for 3 min, followed by sonication for 10 min at 150 W, and 20 kHz (SONOPULS, HD3200) [32]

#### 2.2.6. Kinetic Parameters of Tested Microorganisms

The effect of the active FS on *Salmonella* sp. and *M. luteus* was evaluated by measuring the kinetic parameters, which was associated with the antimicrobial effect. The tested microorganisms were grown in the QP and C biopolymers, C:QP FS, and the FS added with EOs (C:QP:T and C:QP:R). The parameters determined were doubling time (T_d_) and specific growth rate (μ) following the Monod model [33], using the GraphPad Prism 5.0 software (San Diego, CA, USA), showing the active FS effect on microbial growth.

#### 2.2.7. Antimicrobial Activity of Active Filmogenic Suspension

*Salmonella* sp. was activated in TSB broth for 24 h at 37 °C, whereas the same media was used to activate *M. luteus* for 48 h at 30 °C. Then, the FS of C:QP; and FS with EOs (C:QP-R; C:QP-T) were inoculated with each microbial culture to reach 10^6^ CFU/mL. The antimicrobial activity was determined by evaluating the microbial population after 2, 4, 8, 12, 24, and 48 h, using the pour plate method in TSB agar, and incubating as above mentioned.

#### 2.2.8. Filmogenic Suspension Characterization

##### Particle Size

The mean particle diameter of FS was determined with a Zetasizer Nano-ZS laser diffractometer (Malvern Instruments, Worcestershire, UK) at 633 nm and 25 °C, equipped with a backscatter detector [34].

##### Z Potential

Z potential (mV) was determined by phase analysis light scattering (PALS) with a Zetasizer Nano-ZS laser diffractometer (Malvern Instruments, Worcestershire, UK), which determines electrical charge at interface of droplets dispersed in aqueous phase [34].

##### Rheological Properties of Emulsion

A rheometer equipped with concentric cylinder geometry (Discovery Hybrid Rheometer TA Instruments, New Castle, DE, USA), was used to determine the rheological properties of the FS at 25 °C. Temperature equilibration and particles settling were allowed for 5 min before steady-state flow measurements were carried out, using a shear rate range of 0 to 100 s^−1^. Shear stress, shear rate, and apparent viscosity were evaluated using the TRIOS 4 software (TA instruments); the experimental flow curves were fitted to the Casson model (Equation (1)):

(1) $$\sigma^{1/2}=\sigma_{0}^{1/2}+\eta^{1/2}\gamma^{1/2}$$

where σ is shear stress (Pa), σ_0_ is the elastic limit (Pa), γ is the shear rate (s^−1^) and η is apparent viscosity of the fluid [35].

#### 2.2.9. Antimicrobial Evaluation of Filmogenic Suspension

##### Sample Preparation

Samples were prepared according to Mathelié–Guinlet [36] with some modifications. The bacteria were activated for 5 h, at 37 °C, to reach the log phase. Then, 5 µL (10^6^ UFC/mL) of each tested microorganism were inoculated into 2 mL of the FS (control) and 2 mL of FS containing T and R EOs at the previously determined MIC. All mixtures were incubated for 5 h, at 37 °C. An aliquot of 100 µL of each treatment was deposited on glass slides of 26 × 76 mm and 1.1 ± 0.1 mm thick and allowed to dry for 2 h at room temperature in a laminar flow cabinet. Cell adhesion was enhanced by previously adding a layer of 100 µL of FS without EOs to the glass slides and allowed to dry at room temperature.

##### Cells Topography

This determination was carried out using an atomic force microscope (AFM; Multimode V, Veeco, Plainview, NY, USA) in contact mode to avoid any damage to the samples. The images were obtained at a scanning speed from 0.5 to 1.0 Hz, with a resolution of 512 × 512 pixels and at different areas (50 μm × 50 μm; 2.5 μm × 2.5 μm; 0.5 μm × 0.5 μm) [36].

##### Texture Image Analysis

Texture image analysis was conducted following Arzate–Vazquez [37] and was applied to quantitatively characterize the microbial surface microstructure treated with the FS C:QP with and without EOs. Three characteristics were selected: entropy, fractal dimension, and roughness. All images obtained by atomic force microscopy (AFM) were converted to grayscale images. Subsequently, the gray level co-occurrence matrix (GLCM) and differential box count (SDBC) algorithms were applied to obtain the texture characteristics from grayscale images, both included in the Image J 1.52 software (NIH, Bethesda, MD, USA). GLCM is a second-order statistical algorithm that compares two neighboring pixels at once and compiles the frequency with which different gray levels can be found within a restricted area. In this algorithm, three variables are considered: the number of gray levels (0–255), the distance of the pixels (d), and the offset angle (θ). The image texture (entropy) was analyzed by studying the spatial dependence of pixel values represented by a co-occurrence matrix Gd, θ, with the input Gd, θ (i, j), which represents the frequency whereby a pixel with intensity i is adjacent to a pixel with intensity j, separated in direction θ. These parameters were measured at a distance d equal to 1 and an angle equal to 0 °, using Equation (2).

(2) $$\mathrm{Entropy}\;=\;-\overset{}{\underset{ij}{\sum }}\left(G_{d,\theta }\left(i,j\right){\;\log\;(G}_{d,\theta }\left(i,j\right))\right)$$

The fractal texture was evaluated using the power law scale to obtain its fractal dimension (FD) using the SDBC algorithm based on the surface intensity graph. It is generated from 2D grayscale images by plotting pixel coordinates (x, y) versus their gray level in the z-axis. FD was estimated from the slope of the plot log (number of boxes) vs. log (box size) (Equation (3)), where “N” is the number of boxes and “r” is the length of the box size

(3) $$\mathrm{FD}\;=\;\frac{\log\;(N)\;}{\log\;(1/r)}$$

The FD is an object property that shows how much of the space that contains it is occupied and can acquire continuous values in the space of real numbers between 0 and 3. FD tries to measure the extent that a 2D object fills the 3D space, or a one-dimension object resembles a 2D surface allowing the description of the geometry of many natural structures that appear to have great complexity but having the same geometric regularity. According to fractal geometry, the line has FD greater than zero, but less than 1, the FD of a dot is 0, the plane FD is between 1 and 2, whereas that of the cube is between 2 and 3.

FD is directly related to the extent of surface roughness (Rq) (Equation (4)), which was obtained following the protocol of Escamilla–García [12]. Rq is the standard deviation of Zi values indicating roughness (nm); Zi is the difference in the height of i relative to the average height, and N is the number of points in the image.

(4) $$Rq=\sqrt{\frac{\sum {\mathrm{Zi}}^{2}}{N}}$$

#### 2.2.10. Statistical Analysis

All tests were performed in triplicate, and data were evaluated by one-way analysis of variance using GraphPad Prism 5.0 software (GraphPad Software, San Diego, CA, USA). Significant differences were determined by Dunnett’s test, with a significance level of *p* < 0.01. Data are presented as the mean ± standard deviation.

## 3. Results and Discussion

### 3.1. Antimicrobial Activity

**Figure 1:**
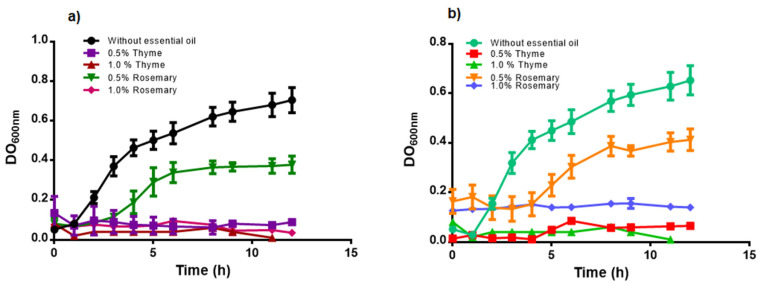
Free essential oils antimicrobial effect on (**a**) *Micrococcus luteus*; (**b**) *Salmonella* sp.

The antimicrobial activity of R and T free EOs against *M. luteus* (Gram-positive) and *Salmonella* sp. (Gram-negative) showed a MIC for both microorganisms of 0.5% (*v*/*v*) and 1.0% (*v*/*v*) of T and R EOs, respectively (Figure 1). The EOs were added to the FS of C:QP using these concentrations.

**Figure 2:**
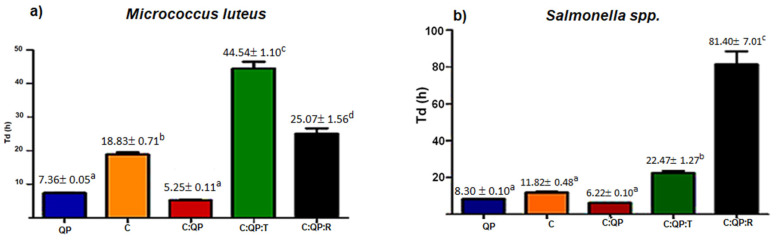
Effect of biopolymers alone, combined, and incorporated with EOs on doubling time of (**a**) *Micrococcus luteus*; (**b**) *Salmonella* sp. T_d_: doubling time; QP: quinoa protein; C: chitosan; T: thyme essential oil 0.5% (*v*/*v*); R: rosemary essential oil 1% (*v*/*v*). Data are presented as the mean ± standard deviation. a–d Indicate significant difference (*p* < 0.001).

The doubling time (T_d_) of *M. luteus* and *Salmonella* sp. is shown in Figure 2. For *M. luteus*, the C:QP FS exhibited the lowest T_d_ value (5.25 ± 0.11 h), but not significantly different from the QP solution (Figure 2a). Solutions of C and FS with added EOs were significantly different from the C:QP FS, with the highest T_d_ obtained by the C:QP:T (44.54 ± 1.10 h), which was 1.77 times the T_d_ of the C:QP:R FS.

The T_d_ value showed by the C solution was lower than those presented by the C:QP:T and C:QP:R FS (Figure 2a), indicating stronger antimicrobial activity of FS added with EOs, than the C solution. *Salmonella* sp. (Figure 2b) was significantly inhibited in the presence of EOs, especially with C:QP:R (81.40 ± 7.01 h), which increased about 3.6 times the T_d_ of C:QP:T (22.47 ± 1.27 h), while the inhibition shown by QP, C, and C:QP FS was significantly lower.

**Figure 3:**
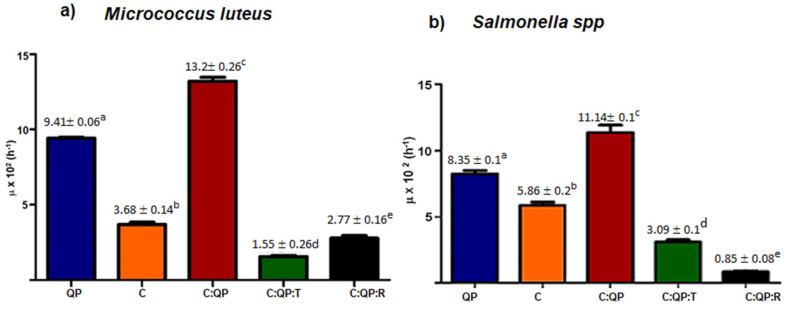
Effect of biopolymers alone, combined, and incorporated with EOs on the specific growth rate of (**a**) *Micrococcus luteus*; (**b**) *Salmonella* sp. μ: specific growth rate; QP: quinoa protein; C: chitosan; T: thyme essential oil 0.5% (*v*/*v*); R: rosemary essential oil 1% (*v*/*v*). Data are presented as the mean ± standard deviation. a–e Indicate significant difference (*p* < 0.01).

The μ value for *M. luteus* decreased in the presence of the FS C:QP:T and C:QP:R (Figure 3a). The C:QP:T FS presented the lowest μ value (5.55 ± 0.07 h^−1^), indicating higher antimicrobial activity of C:QP:T than that of C:QP:R, whereas the highest microbial growth was shown by the C:QP FS (μ = 13.2 ± 0.26 h^−1^) (Figure 3a). All FS resulted in significantly different *Salmonella* sp. specific growth rate (Figure 3b). Both EOs in the FS showed high *Salmonella* sp. inhibition, but the FS containing R EO showed the highest (μ = 0.085 ± 0.001 h^−1^). The FS producing the least inhibition was C:QP (μ = 0.111 ± 0.001 h^−1^).

Although FS added with T EO showed antimicrobial activity against both bacteria, the effect was greater against *M. luteus*, which is reflected in a doubling time about twice of that showed for *Salmonella* sp. (T_d_ = 22.47 ± 1.27 h) (Figure 3). Hosseini [38] reported that T EO had greater activity against Gram-positive than Gram-negative bacteria due to their thick layer of peptidoglycan (90–95%) and the presence of an outer lipopolysaccharide membrane [39]. The outer membrane has also been associated with higher antimicrobial activity of the combination of C and EOs because it restricts hydrophobic compounds [21].

However, the effectiveness of EOs in the presence of C depends on their composition, structure, and functional groups. The C:QP:R FS showed strong *Salmonella* sp. inhibition, with T_d_ = 81.04 ± 7.01 h, value 3.23 times greater than that of *M. luteus*.

**Figure 4:**
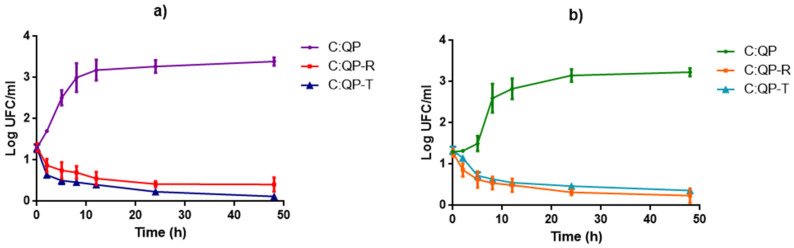
Antimicrobial activity of filmogenic suspensions with and without thyme and rosemary essential oils. (**a**) *Micrococcus luteus*, (**b**) *Salmonella* sp.

The FS C:QP did not exhibit inhibition against *M. luteus*, but FS with EOs exerted an antimicrobial effect (Figure 4a). There was 2 log population reduction exerted by FS containing either T or R EOs after 4 h of contact time, whereas after 24 h, the reduction increased to 3 log cycles. In contrast, *Salmonella* sp. population was reduced by the FS containing either R or T EOs about 1 log cycle after 2 h, whereas the maximum reduction of 3 log cycles was achieved after 48 h (Figure 4b). As expected, higher inhibition was observed against the Gram-positive bacterium *M. luteus*.

### 3.2. Filmogenic Suspension Characterization

#### 3.2.1. Stability

The stability of FS was evaluated by particle size, Z potential, and polydispersity index (IPD). Z potential values were evaluated initially and after 10 d of storage (Table 1), giving values in the range −46.21 to −54.13 mV. After 10 d of storage, the emulsion C:QP:R significantly increased its z potential, whereas all FS showed increased values, although not significant.

Absolute values of Z-potential greater than 30 mV indicate high emulsions stability [40], and thus, all the emulsions are stable. This effect may be associated with the presence of the non-ionic surfactant (Tween 80), which permitted a balance of what is known as hydrophilic-lipophilic numbness, allowing the maintenance of stable emulsions, even during storage [41].

The negative charge of the Z potential was attributed to the alkaline pH used to produce the emulsions. Despite chitosan positive charges, aqueous FS at pH > 6 promotes a higher number of deprotonated species followed by aggregation due to hydrogen bonds formation involving the neutralized NH_2_ groups of the chitosan chains [40]. In addition, a study has shown that Tween can favor negative electrical charges, which are attributed to the presence of anionic impurities [42]. The stability of FS may be enhanced by the resulting protein network, which, through hydrophilic and hydrophobic groups, allows both water and oil interactions, preventing the FS from collapsing [43].

From the results obtained, the particle size presented two main populations for each sample. It was observed that the population is representing agglomerated particles comprised most of the FS (Table 2). The FS with added EOs showed significantly increased particle size, especially considering the more abundant large particles (597.56 ± 37.59 nm to 677.02 ± 35.08 nm) as compared to FS without EOs. However, there was no significant difference among the smaller particles (97.55 ± 10.79 nm to 153.58 ± 13.76 nm). On the other hand, the addition of EOs generated a significant increase in the polydispersity index (PDI).

PDI is the ratio of weight average to number average molecular weight, and values close to zero indicate highly monodisperse (homogeneous) FS, while values > 0.7 indicate a broad particle size distribution [44]. The results in Table 2 show PDI values between 0.5 and 0.80, suggesting that the produced FS are heterogeneous, while EOs addition caused greater heterogeneity. Quinoa protein is soluble at pH 11, where it has greater solubility and water absorption capacity. It also contains high amounts of sulfur amino acids, threonine, and tryptophan, although experiencing small protein denaturation [45]. FS were prepared using this pH, which favored the formation of aggregates, which was reflected in suspensions exhibiting two different particle sizes (Table 2). The C:QP FS revealed one population of particle sizes close to 600 nm, which may correspond to protein aggregates resulting from the formation of hydrogen bonds within the QP and with C, together with hydrophobic interactions and covalent disulfide bonds that are formed in the protein under these conditions [46]. Disulfide bond formation is favored by the presence of TG and by heat processing of the FS, leading to the formation of agglomerates [46]. The particle size of the FS (597.56 ± 37.59 nm) increased in the presence of EOs, which was attributed to the increase in hydrophobic interactions among drops of individual EOs [47].

#### 3.2.2. Rheology

The FS rheology was successfully fitted to the Casson model (R^2′^ = 0.995–0.998), without significant differences (Table 3). The C:PQ-R FS showed the lowest elastic limit but was not significantly different from the other emulsions. Similarly, the calculated apparent viscosity of the different FS was not significantly different.

Non-Newtonian fluids that behave as elastic solids and show shear-thinning behavior can be fitted with the Casson’s rheological model, which shows infinite viscosity at zero shear rate [48].

The FS made with C:PQ-R showed the highest apparent viscosity (3.04 ± 0.002 × 10^−3^ Pa.s), which according to Dapueto [49], can be related to the formation of significant amounts of protein aggregates, associated with the increased particle sizes in the presence of EOs. Proteins, like surfactants, form monolayers in aqueous solutions that can have high elastic properties behaving like sticky droplets [43], whose interactions with the EOs promote elasticity reduction.

**Figure 5:**
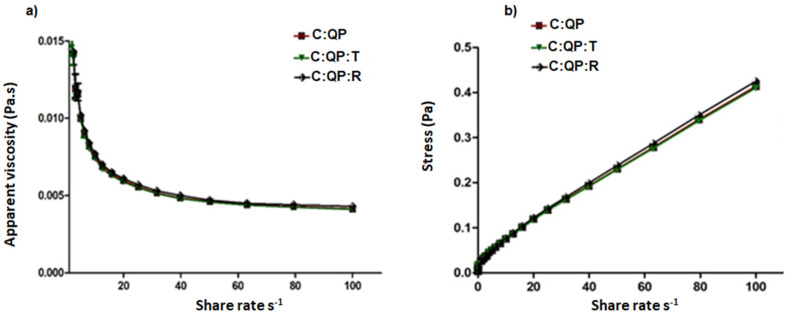
Rheology of filmogenic suspensions C:QP, C:QP:T, and C:QP:R. (**a**) Apparent viscosity vs. shear rate; (**b**) Stress vs. shear rate. QP: quinoa protein; C: chitosan; T: essential oil of thyme; A: rosemary essential oil. Data are presented as the mean ± standard deviation.

The apparent viscosity of FS decreased when applying higher shear rates (Figure 5a). However, when reaching a speed of approximately 20 s^−1^, as the shear rate increased, there were few changes in apparent viscosity.

Figure 5b shows a directly proportional relationship between stress and shear rate for the FS. However, for both properties, the evaluated FS were not significantly different.

#### 3.2.3. Antimicrobial Evaluation of Filmogenic Suspension by Atomic Force Microscopy

**Figure 6:**
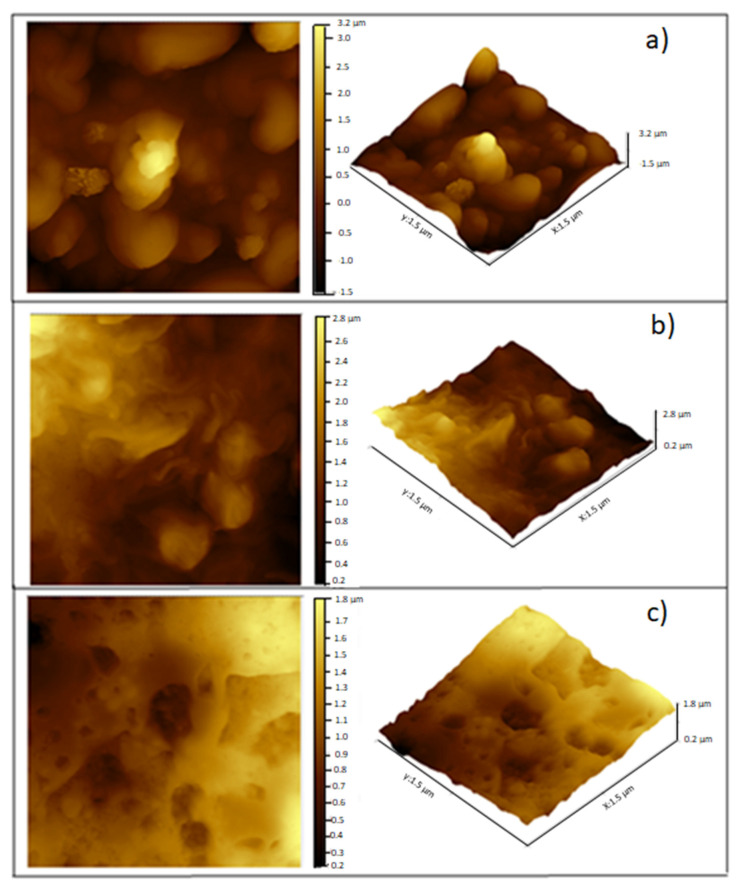
Visualization by AFM of the antimicrobial effect on *Micrococcus luteus* of EOs incorporated into the filmogenic suspension. (**a**) Control, (**b**) Rosemary essential oil, (**c**) Thyme essential oil.

Figure 6 shows *M. luteus* topography for the control and FS with EOs. *M. luteus* revealed a coccus morphology when growing in the FS without EOs (Figure 6a). However, in the presence of the R EO (Figure 6b) and T EO (Figure 6c), *M. luteus* lost its characteristic spherical structure, suggesting an antimicrobial effect. The T EO showed a greater antimicrobial effect on *M. luteus* by causing massive destruction of its structure. Meanwhile, the effect of R EO was milder because some cells retained their spherical shape, suggesting partial inhibition, as shown in Figure 1.

**Figure 7:**
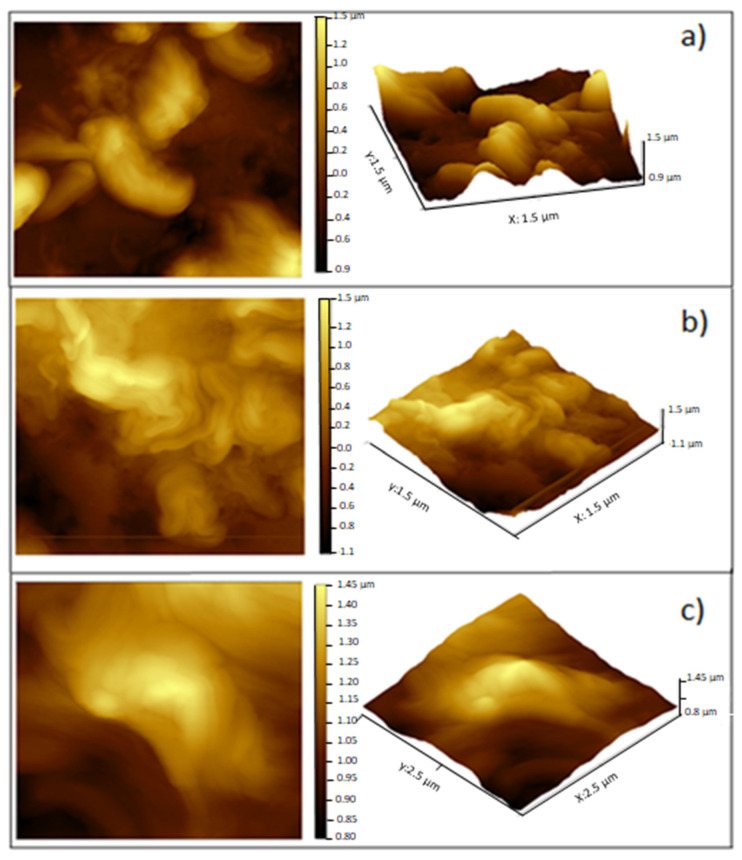
Visualization by AFM of the antimicrobial effect on *Salmonella* sp. of EOs incorporated into the filmogenic suspension. (**a**) Control, (**b**) Rosemary essential oil, (**c**) Thyme essential oil.

*Salmonella* sp. is characterized by presenting the bacillus shape (Figure 7a). The R EO (Figure 7b) and T EO (Figure 7c) exerted an antimicrobial effect on this microorganism by damaging the cell surface. The EOs showed antimicrobial activity against the tested microorganisms (Figure 1), and when incorporated into the FS, they modified the growth kinetic parameters (Figure 2 and Figure 3), exerting antimicrobial effect (Figure 4b), which was visualized by using AFM.

The control treatments exhibited rough and heterogeneous surfaces consisting of agglomerated matter with a structure that coincided with the morphologies of *M. luteus* and *Salmonella* sp. The spherical shape of *M. luteus* showed dimensions of 0.5–3.5 µm (Figure 6a), whereas *Salmonella* sp. revealed a short bar geometry (Figure 7a) [50,51]. However, when adding EOs, these shapes were lost, which was attributed to damage to the cell wall and cytoplasmic membrane with loss of structural integrity, as confirmed by the population reduction (Figure 4). This effect might include the disruption of the proton motive force, inhibition of substrate oxidation, and disruption of DNA synthesis [52]. The hydrophobicity of essential oils enables the separation of lipids present in the cell membrane of bacteria, altering their structure, making them more permeable, and causing cell lysis [53].

#### 3.2.4. Image Analysis

Entropy (E) is a measure of the heterogeneity of the images, and to quantitatively evaluate the images obtained by AFM; the grayscale co-occurrence matrix algorithm was used. In addition, the fractal dimension (FD), also known as fractal texture through the Differential Box Count (SDBC) algorithm, is a parameter related to the irregularity of the surface.

**Figure 8:**
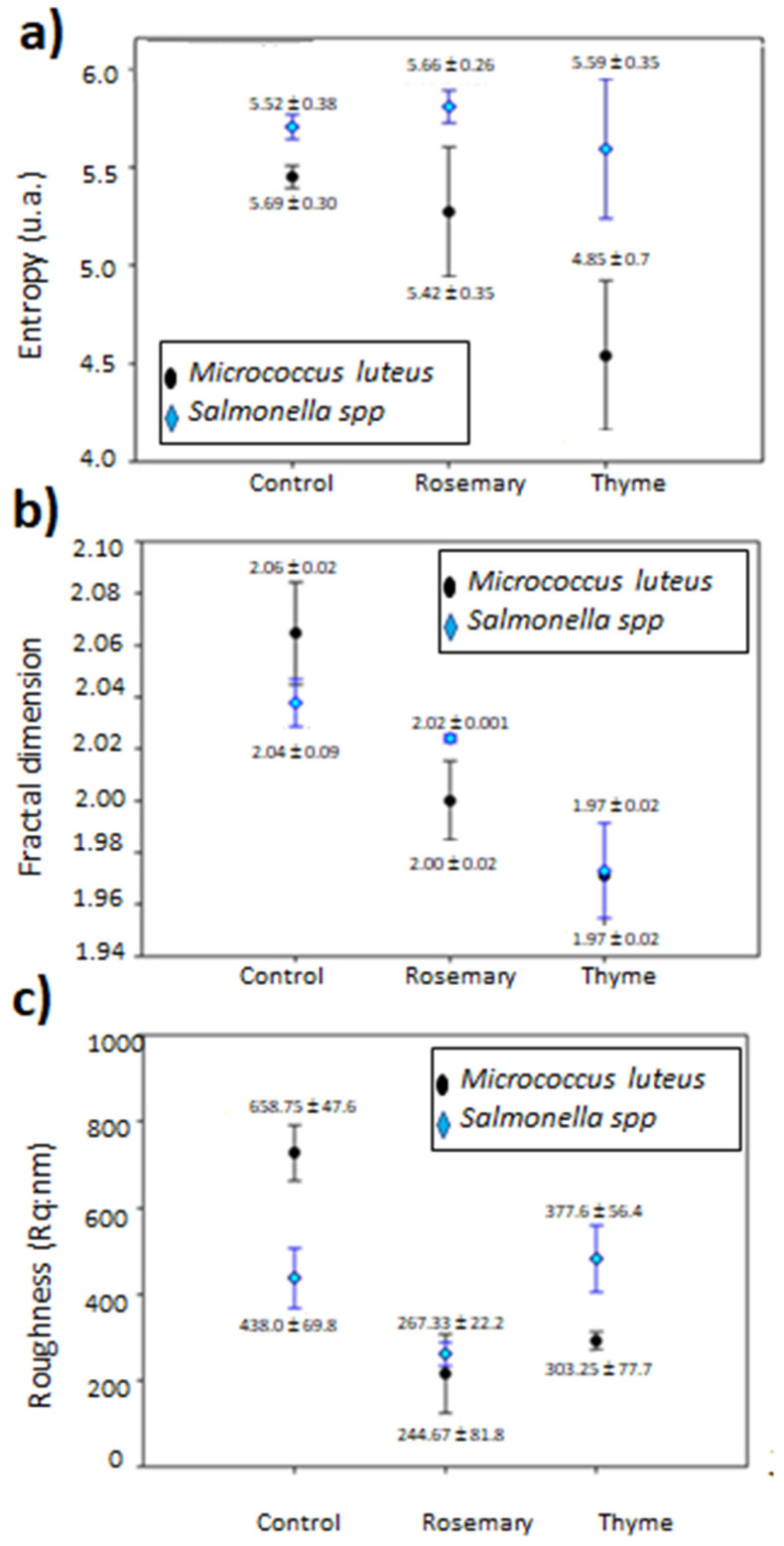
Atomic force micrographs image analysis on the effect of filmogenic suspensions with and without essential oils of *Salmonella* sp. and *M. luteus.* (**a**) Entropy (u.a: arbitrary units), (**b**) fractal dimension, and (**c**) roughness.

There are no reports about the E and FD calculated from image analysis evaluation to confirm the antimicrobial effect of T and R EOs on the two microorganisms used in this work, as shown in Figure 4. From Figure 8a, it is observed that the entropy of the samples of C:QP did not significantly change in the FS, incorporating the EOs when *Salmonella* sp. was inoculated.

The lowest entropy was observed for *M. luteus* in T EO suspensions (Figure 8a), suggesting a more homogeneous image relative to the control, which was attributed to the effect of microbial growth inhibition, which correlates well with the antimicrobial activity shown in Figure 4a. The fractal dimension (Figure 8b) decreased when EOs were added, and this phenomenon was observed in the roughness value (Figure 8c), except for *Salmonella* sp. in the presence of T EO, which may be associated with cell lysis caused by the EOs.

According to our results, the FD decreased in the presence of EOs. Fractal dimension is a mathematical concept in which a set of multiple scales exhibits the same repeating pattern on each scale, which can be transferred to texture analysis. This parameter is correlated with roughness; the larger the FD, the rougher the texture [53]. The addition of EOs to the C:QP FS generally produced smoother surfaces, and thus, the roughness reduction was attributed to cell lysis promoted by the EOs.

The characteristic structures of *M. luteus* and *Salmonella* sp. provided high FD and entropy, considering it as the randomness or degree of disorder showed by the image; whereas the roughness was high for *M. luteus,* but lower for *Salmonella* sp.

The entropy is higher when all elements of the co-occurrence matrix are equal and smaller when the elements are different [54]; thus, more homogeneous surfaces are probably the result of the antimicrobial effect of EOs.

## 4. Conclusions

Essential oils imparted antimicrobial activity on *M. luteus* and *Salmonella* sp., at concentrations of 0.5% (*v*/*v*) and 1% *(v*/*v*) for thyme and rosemary EOs, respectively. When EOs were incorporated into the FS, thyme EO showed a greater inhibitory effect on *Salmonella* sp. and *M. luteus* than rosemary EO.

The FS of C:QP with and without EOs were stable but heterogeneous dispersions. EOs addition significantly increased the particle size distribution, showing two major populations. Casson’s rheological model was successfully fitted to the non-Newtonian fluids (FS with and without EOs), that behave like an elastic solid. Formation of protein aggregates allowed greater interaction between protein and aqueous phase, which increased the apparent viscosity of the C:QP-R FS, compared to the control.

AFM permitted the evaluation of the characteristic structures of *M. luteus* and *Salmonella* sp. and confirmed the antimicrobial activity of the EOs, which was also monitored by significant changes in their kinetic parameters. Image analysis of FS containing T and R EOs showed that entropy, roughness, and FD changes might correlate with the generation of more homogeneous surfaces and with the antimicrobial effect. The properties of the active FS may be of use as an alternative coating material for food preservation.

## Figures and Tables

**Table 1:** Z potential of filmogenic suspensions.

Z Potential (mV)		
Sample	Day 0	Day 10
C:QP	−47.69 ± 3.19 ^ab^	−50.60 ± 4.04 ^ab^
C:QP:T	−46.86 ± 4.38 ^ab^	−52.01 ± 4.04 ^ab^
C:QP:R	−46.21 ± 3.83 ^a^	−54.13 ± 4.73 ^b^

QP: Quinoa protein; C: Chitosan; T: thyme essential oil, R: rosemary essential oil. Mean values in the same column showing the same lowercase letter are not significantly different (*p* < 0.01).

**Table 2:** Particle size and polydispersity index of filmogenic suspensions.

Sample	Particle Size (nm)	Particle Size Intensity (%)	PDI
C:QP	597.56 ± 37.59 ^a^	89.41 ± 2.66 ^a^	0.51 ± 0.07 ^a^
	97.55 ± 10.79 ^b^	7.28 ± 0.49 ^b^	
C:QP:T	677.02 ± 50.34 ^c^	73.35 ± 4.16 ^c^	0.80 ± 0.04 ^b^
	153.58 ± 13.76 ^b^	22.04 ± 2.60 ^d^	
C:QP:R	672.98 ± 35.08 ^c^	79.79 ± 3.74 ^e^	0.79 ± 0.05 ^b^
	120.93 ± 20.96 ^b^	16.17 ± 3.04 ^d^	

QP: Quinoa protein; C: Chitosan; T: thyme essential oil, R: rosemary essential oil. Mean values in the same column showing the same lowercase letter are not significantly different (*p* < 0.01).

**Table 3:** Rheological properties of filmogenic suspensions.

Sample	σ_o_ (×10^3^ Pa)	η (×10^3^ Pa.s)	R^2^
PQ:C	11.23 ± 1.42 ^a^	2.82 ± 0.15 ^a^	0.997
C:PQ–T	11.51 ± 0.50 ^a^	2.80 ± 0.02 ^ab^	0.995
C:PQ–R	9.96 ± 0.24 ^a^	3.04 ± 0.002 ^ac^	0.998

σ_o_: Elastic limit; η: Apparent viscosity; QP: Quinoa protein; C: Chitosan; T: thyme essential oil, R: rosemary essential oil. Mean values in the same column showing the same lowercase letter are not significantly different (*p* < 0.01).

## References

[1] Valde F.D., Banos S.B., Valdes D.F., Ramirez A.O., García P.A., Falcón R.A. (2015). Películas y recubrimientos comestibles: Una alternativa favorable en la conservación poscosecha de frutas y hortalizas. Rev. Cien. Tec. Agros..

[2] Guerreiro T.M., De Oliveira D.N., Melo C.F.O.R., Lima E.D.O., Catharino R.R. (2018). Migration from plastic packaging into meat. Food Res. Int..

[3] Majid I., Nayik G.A., Dar S.M., Nanda V. (2018). Novel food packaging technologies: Innovations and future prospective. J. Saudi Soc. Agric. Sci..

[4] Ahmed I., Lin H., Zou L., Brody A.L., Li Z., Qazi I.M., Pavase T.R., Lv L. (2017). A comprehensive review on the application of active packaging technologies to muscle foods. Food Control.

[5] Escamilla-García M., Calderón-Domínguez G., Chanona-Pérez J., Mendoza-Madrigal A.G., Di Pierro P., García-Almendárez B.E., Amaro-Reyes A., Regalado-González C. (2017). Physical, structural, barrier, and antifungal characterization of chitosan–zein edible films with added essential oils. Int. J. Mol. Sci..

[6] Thakur R., Pristijono P., Bowyer M., Singh S.P., Scarlett C.J., Stathopoulos C.E., Vuong Q.V. (2019). A starch edible surface coating delays banana fruit ripening. LWT.

[7] Dehghani S., Hosseini S.V., Regenstein J.M. (2018). Edible films and coatings in seafood preservation: A review. Food Chem..

[8] Elsabee M.Z., Abdou E.S. (2013). Chitosan based edible films and coatings: A review. Mater. Sci. Eng. C.

[9] Jost V., Kobsik K., Schmid M., Noller K. (2014). Influence of plasticiser on the barrier, mechanical and grease resistance properties of alginate cast films. Carbohydr. Polym..

[10] Ma Z., Garrido-Maestu A., Jeong K.C. (2017). Application, mode of action, and *in vivo* activity of chitosan and its micro- and nanoparticles as antimicrobial agents: A review. Carbohydr. Polym..

[11] Enujiugha V.N., Oyinloye A.M. (2019). Protein-Lipid Interactions and the Formation of Edible Films and Coatings. Encyclopedia of Food Chemistry.

[12] Escamilla-García M., Delgado-Sánchez L.F., Ríos-Romo R.A., García-Almendárez B.E., Calderón-Domínguez G., Méndez-Méndez J.V., Amaro-Reyes A., Di Pierro P., Regalado-González C. (2019). Effect of transglutaminase cross-linking in protein isolates from a mixture of two quinoa varieties with chitosan on the physicochemical properties of edible films. Coatings.

[13] Caro N., Medina E., Díaz-Dosque M., López L., Abugoch L., Tapia C. (2016). Novel active packaging based on films of chitosan and chitosan/quinoa protein printed with chitosan-tripolyphosphate-thymol nanoparticles via thermal ink-jet printing. Food Hydrocoll..

[14] Musumeci T., Puglisi G., Pignatello R. (2013). 10. Antimicrobial agents. Drug-Biomembrane Interaction Studies.

[15] El Asbahani A., Miladi K., Badri W., Sala M., Addi E.H.A., Casabianca H., El Mousadik A., Hartmann D., Jilale A., Renaud F.N.R. (2015). Essential oils: From extraction to encapsulation. Int. J. Pharm..

[16] Donsì F., Ferrari G. (2016). Essential oil nanoemulsions as antimicrobial agents in food. J. Biotechnol..

[17] Benjemaa M., Neves M.A., Falleh H., Isoda H., Ksouri R., Nakajima M. (2018). Nanoencapsulation of *Thymus capitatus* essential oil: Formulation process, physical stability characterization and antibacterial efficiency monitoring. Ind. Crop. Prod..

[18] Gonçalves N.D., Pena F.D.L., Sartoratto A., Derlamelina C., Duarte M.C.T., Antunes A.E.C., Prata A.S. (2017). Encapsulated thyme (*Thymus vulgaris* ) essential oil used as a natural preservative in bakery product. Food Res. Int..

[19] Grande-Tovar C.D., Chaves-Lopez C., Serio A., Rossi C., Paparella A. (2018). Chitosan coatings enriched with essential oils: Effects on fungi involved in fruit decay and mechanisms of action. Trends Food Sci. Technol..

[20] Sadekuzzaman M., Mizan F.R., Kim H.-S., Yang S., Ha S.-D. (2018). Activity of thyme and tea tree essential oils against selected foodborne pathogens in biofilms on abiotic surfaces. LWT.

[21] Yuan G., Chen X., Li D. (2016). Chitosan films and coatings containing essential oils: The antioxidant and antimicrobial activity, and application in food systems. Food Res. Int..

[22] Turasan H., Sahin S., Sumnu G. (2015). Encapsulation of rosemary essential oil. LWT.

[23] Rizzo V., Amoroso L., Licciardello F., Mazzaglia A., Muratore G., Restuccia C., Lombardo S., Pandino G., Strano M.G., Mauromicale G. (2018). The effect of *sous vide* packaging with rosemary essential oil on storage quality of fresh-cut potato. LWT.

[24] Okoh O., Sadimenko A., Afolayan A.J. (2010). Comparative evaluation of the antibacterial activities of the essential oils of *Rosmarinus officinalis* L. obtained by hydrodistillation and solvent free microwave extraction methods. Food Chem..

[25] Souza V.G., Pires J.R.A., Vieira É.T., Coelhoso I.M., Duarte M.P., Fernando A.L. (2019). Activity of chitosan-montmorillonite bionanocomposites incorporated with rosemary essential oil: From *in vitro* assays to application in fresh poultry meat. Food Hydrocoll..

[26] Tack D.M., Ray L., Griffin P.M., Cieslak P.R., Dunn J., Rissman T., Jervis R., Lathrop S., Muse A., Duwell M. (2020). Preliminary incidence and trends of infections with pathogens transmitted commonly through food—Foodborne Diseases Active Surveillance Network, 10 U.S. Sites, 2016–2019. MMWR Morb. Mortal. Wkly. Rep..

[27] Elsohaimy S., Refaay T., Zaytoun M. (2015). Physicochemical and functional properties of quinoa protein isolate. Ann. Agric. Sci..

[28] Ruiz G.A., Xiao W., Van Boekel M., Minor M., Stieger M. (2016). Effect of extraction pH on heat-induced aggregation, gelation and microstructure of protein isolate from quinoa (*Chenopodium quinoa* Willd). Food Chem..

[29] Giosafatto C., Fusco A., Al-Asmar A., Mariniello L. (2020). Microbial transglutaminase as a tool to improve the features of hydrocolloid-based bioplastics. Int. J. Mol. Sci..

[30] E Jensen S., Campbell J.N. (1976). Peptidoglycan biosynthesis in *Micrococcus luteus* (sodonensis): Transglycosidase and phosphodiesterase activities in membrane preparations. J. Bacteriol..

[31] Deng L., Alexander A.A., Lei S., Anderson J.S. (2010). The cell wall teichuronic acid synthetase (TUAS) is an enzyme complex located in the cytoplasmic membrane of *Micrococcus luteus*. Biochem. Res. Int..

[32] Di Pierro P., Marquez G.R., Mariniello L., Sorrentino A., Villalonga R., Porta R. (2013). Effect of transglutaminase on the mechanical and barrier properties of whey protein/pectin films prepared at complexation pH. J. Agric. Food Chem..

[33] Olivares-Marin I.K., González-Hernádez J.C., Regalado-González C., Madrigal-Pérez L.A. *Saccharomyces cerevisiae* exponential growth kinetics in batch culture to analyze respiratory and fermentative metabolism. https://pubmed.ncbi.nlm.nih.gov/30320748/.

[34] Arredondo-Ochoa T., García-Almendárez B.E., Escamilla-García M., Martín-Belloso O., Rossi-Márquez G., Medina-Torres L., Regalado-González C. (2017). Physicochemical and antimicrobial characterization of beeswax–starch food-grade nanoemulsions incorporating natural antimicrobials. Int. J. Mol. Sci..

[35] Zhao X., Liu F., Ma C., Yuan F., Gao Y. (2015). Effect of carrier oils on the physicochemical properties of orange oil beverage emulsions. Food Res. Int..

[36] Mathelié-Guinlet M., Grauby-Heywang C., Martin A., Février H., Moroté F., Vilquin A., Béven L., Delville M.-H., Cohen-Bouhacina T. (2018). Detrimental impact of silica nanoparticles on the nanomechanical properties of *Escherichia coli*, studied by AFM. J. Colloid Interface Sci..

[37] Arzate-Vázquez I., Chanona-Pérez J., Calderón-Domínguez G., Térres-Rojas E., Garibay-Febles V., Martínez-Rivas A., Gutierrez-López G.F. (2012). Microstructural characterization of chitosan and alginate films by microscopy techniques and texture image analysis. Carbohydr. Polym..

[38] Hosseini M., Razavi S., Mousavi M. (2009). Antimicrobial, physical and mechanical properties of chitosan-based films incorporated with thyme, clove and cinnamon essential oils. J. Food Process. Preserv..

[39] Khorshidian N., Yousefi M., Khanniri E., Mortazavian A.M. (2018). Potential application of essential oils as antimicrobial preservatives in cheese. Innov. Food Sci. Emerg. Technol..

[40] Roy J.C., Salaün F., Giraud S., Ferri A., Guan J. (2017). Surface behavior and bulk properties of aqueous chitosan and type-B gelatin solutions for effective emulsion formulation. Carbohydr. Polym..

[41] Hong I.K., Kim S.I., Lee S.B. (2018). Effects of HLB value on oil-in-water emulsions: Droplet size, rheological behavior, zeta-potential, and creaming index. J. Ind. Eng. Chem..

[42] Guerra-Rosas M.I., Morales-Castro J., Ochoa-Martínez L.A., Salvia-Trujillo L., Martín-Belloso O. (2016). Long-term stability of food-grade nanoemulsions from high methoxyl pectin containing essential oils. Food Hydrocoll..

[43] Hoffmann H., Reger M. (2014). Emulsions with unique properties from proteins as emulsifiers. Adv. Colloid Interface Sci..

[44] Danaei M., Dehghankhold M., Ataei S., Davarani F.H., Javanmard R., Dokhani A., Khorasani S., Mozafari M.R. (2018). Impact of particle size and polydispersity index on the clinical applications of lipidic nanocarrier systems. Pharmaceutics.

[45] Dakhili S., Abdolalizadeh L., Hosseini S.M., Shojaee-Aliabadi S., Mirmoghtadaie L. (2019). Quinoa protein: Composition, structure and functional properties. Food Chem..

[46] Wu X., Liu Y., Liu A., Wang W. (2017). Improved thermal-stability and mechanical properties of type I collagen by cross-linking with casein, keratin and soy protein isolate using transglutaminase. Int. J. Biol. Macromol..

[47] Yang M., Liu F., Tang C.-H. (2013). Properties and microstructure of transglutaminase-set soy protein-stabilized emulsion gels. Food Res. Int..

[48] Animasaun I., Adebile E., Fagbade A. (2016). Casson fluid flow with variable thermo-physical property along exponentially stretching sheet with suction and exponentially decaying internal heat generation using the homotopy analysis method. J. Niger. Math. Soc..

[49] Dapueto N., Troncoso E., Mella C., Zúñiga R. (2019). The effect of denaturation degree of protein on the microstructure, rheology and physical stability of oil-in-water (O/W) emulsions stabilized by whey protein isolate. J. Food Eng..

[50] Hafedh H., Fethi B.A., Mejdi S., Emira N., Amina B. (2010). Effect of *Mentha longifolia* L. ssp longifolia essential oil on the morphology of four pathogenic bacteria visualized by atomic force microscopy. Afr. J. Microbiol. Res..

[51] Liu Y., Mollaeian K., Ren J. (2019). Finite element modeling of living cells for AFM indentation-based biomechanical characterization. Micron.

[52] Zengin H., Baysal A.H. (2014). Antibacterial and antioxidant activity of essential oil terpenes against pathogenic and spoilage-forming bacteria and cell structure-activity relationships evaluated by SEM microscopy. Molecules.

[53] Di Cataldo S., Ficarra E. (2017). Mining textural knowledge in biological images: Applications, methods and trends. Comput. Struct. Biotechnol. J..

[54] Reis M.M., Van Beers R., Al-Sarayreh M., Shorten P., Yan W.Q., Saeys W., Klette R., Craigie C. (2018). Chemometrics and hyperspectral imaging applied to assessment of chemical, textural and structural characteristics of meat. Meat Sci..

